# Supplementary material for: Anatomical and physiological responses of Aechmea blanchetiana (Bromeliaceae) induced by silicon and sodium chloride stress during in vitro culture
Source: PeerJ. 2023 Jan 11;11:e14624. doi: 10.7717/peerj.14624 (PMC9840392; doi:10.7717/peerj.14624)
Supplement: Supplemental Information 2 [file peerj-11-14624-s002.docx]

**Supplement 1** Summarized ANOVA results for all characteristics including total degrees of freedom and *p* values for the variation factors (Si and NaCl)

| Variable | Degrees of freedom | *p* values | | |
| --- | --- | --- | --- | --- |
|  |  | Si | NaCl | Si x NaCl |
| Number of metaxylem vessels (roots) | 47 | 0.4932 | 0.9861 | 0.8001 |
| Root diameter (μm) | 47 | 0.1565 | 0.0027 | 0.9195 |
| Thickness of cell walls in the exodermis (μm) | 47 | 0.0000 | 0.0477 | 0.0009 |
| Stomatal density at the base (0.01 mm^-2^) | 47 | 0.6097 | 0.0112 | 0.8644 |
| Stomatal density at the middle (0.01 mm^-2^) | 47 | 0.0013 | 0.9193 | 0.9371 |
| Size of the stomata (μm^2^) | 47 | 0.0442 | 0.0678 | 0.2110 |
| Thickness of the adaxial faces of the epidermis (µm) | 47 | 0.1527 | 0.0000 | 0.9427 |
| Thickness of the abaxial faces of the epidermis (µm) | 47 | 0.4660 | 0.0005 | 0.1646 |
| Thickness of the chlorenchyma (μm) | 47 | 0.2411 | 0.0354 | 0.1247 |
| Area of sclerenchyma (μm^2^) | 47 | 0.6658 | 0.1158 | 0.2406 |
| Area of phloem (μm^2^) | 47 | 0.4863 | 0.1176 | 0.1574 |
| Number of xylem vessels | 47 | 0.0086 | 0.0026 | 0.0015 |
| Diameter of xylem vessels (μm) | 47 | 0.5671 | 0.1665 | 0.9360 |
| SOD (U min^-1^ mg^-1^ prtn) leaves | 39 | 0.0000 | 0.0000 | 0.0004 |
| SOD (U min^-1^ mg^-1^ prtn) root | 39 | 0.0000 | 0.0000 | 0.0017 |
| CAT (µmol min^-1^ mg^-1^ prtn) leaves | 39 | 0.0000 | 0.0007 | 0.0018 |
| CAT (µmol min^-1^ mg^-1^ prtn) root | 39 | 0.5596 | 0.0000 | 0.0340 |
| APX (nmol min^-1^ mg^-1^ prtn) leaves | 39 | 0.0001 | 0.0000 | 0.2495 |
| APX (nmol min^-1^ mg^-1^ prtn) root | 39 | 0.0000 | 0.0000 | 0.1405 |
| Chl *a* | 63 | 0.0006 | 0.3142 | 0.1464 |
| Chl b | 63 | 0.6908 | 0.2424 | 0.3231 |
| Car | 63 | 0.0001 | 0.6295 | 0.3180 |
| Chl *a/b* | 63 | 0.2286 | 0.1034 | 0.6724 |
| Chl total | 63 | 0.0130 | 0.2566 | 0.1507 |
| ΦPSII | 95 | 0.0000 | 0.0035 | 0.0772 |
| ETR | 95 | 0.0000 | 0.0046 | 0.0698 |
| NPQ | 95 | 0.0006 | 0.3685 | 0.2628 |
| F_V_/F_M_ | 95 | 0.0097 | 0.0639 | 0.0684 |
| qP | 95 | 0.0000 | 0.0016 | 0.0004 |
| qN | 95 | 0.0000 | 0.1098 | 0.0004 |
| qL | 95 | 0.0000 | 0.0033 | 0.0000 |
| ΦNO | 95 | 0.0007 | 0.0641 | 0.0010 |
| ΦNPQ | 95 | 0.0000 | 0.0416 | 0.0105 |
| S (g Kg^-1^) | 23 | 0.0214 | 0.0004 | 0.0200 |
| Mg (g Kg^-1^) | 23 | 0.0000 | 0.0000 | 0.0092 |
| B (mg Kg^-1^) | 23 | 0.0537 | 0.1853 | 0.0254 |
| Na (mg Kg^-1^) | 23 | 0.0004 | 0.0000 | 0.0007 |
| Fe (mg Kg^-1^) | 23 | 0.9143 | 0.0150 | 0.0712 |
| Zn (mg Kg^-1^) | 23 | 0.0325 | 0.0030 | 0.1362 |
| Mn (mg Kg^-1^) | 23 | 0.0093 | 0.0021 | 0.1878 |
| K (g Kg^-1^) | 23 | 0.0622 | 0.2076 | 0.0731 |
| N (g Kg^-1^) | 23 | 0.0220 | 0.2334 | 0.4060 |
| Ca (g Kg^-1^) | 23 | 0.0008 | 0.0001 | 0.1037 |
| Na /K (g Kg^-1^) | 23 | 0.0442 | 0.0000 | 0.1295 |
| Fresh weight of aerial part (g plant^-1^) | 39 | 0.0008 | 0.0052 | 0.0024 |
| Fresh weight of roots (g plant^-1^) | 39 | 0.2689 | 0.0000 | 0.0000 |
